# The extracytoplasmic function sigma factor σ^VreI^ is active during infection and contributes to phosphate starvation-induced virulence of *Pseudomonas aeruginosa*

**DOI:** 10.1038/s41598-020-60197-x

**Published:** 2020-02-21

**Authors:** Joaquín R. Otero-Asman, José M. Quesada, Kin K. Jim, Alain Ocampo-Sosa, Cristina Civantos, Wilbert Bitter, María A. Llamas

**Affiliations:** 1 Department of Environmental Protection, Estación Experimental del Zaidín-Consejo Superior de Investigaciones Científicas, Granada, Spain; 2 Department of Medical Microbiology and Infection Control, Amsterdam University medical centers, location VU University, Amsterdam, The Netherlands; 3 Service of Microbiology, Hospital Universitario Marqués de Valdecilla-Instituto de Investigación Sanitaria Valdecilla, Santander, Spain

**Keywords:** Bacterial pathogenesis, Pathogens

## Abstract

The extracytoplasmic function sigma factor σ^VreI^ of the human pathogen *Pseudomonas aeruginosa* promotes transcription of potential virulence determinants, including secretion systems and secreted proteins. Its activity is modulated by the VreR anti-σ factor that inhibits the binding of σ^VreI^ to the RNA polymerase in the absence of a (still unknown) inducing signal. The *vreI-vreR* genes are expressed under inorganic phosphate (Pi) starvation, a physiological condition often encountered in the host that increases *P. aeruginosa* pathogenicity. However, whether or not σ^VreI^ is active *in vivo* during infection and contributes to the Pi starvation-induced virulence of this pathogen has not been analyzed yet. Using zebrafish embryos and a human alveolar basal epithelial cell line as *P. aeruginosa* hosts, we demonstrate in this work that σ^VreI^ is active during infection and that lack of σ^VreI^ considerably reduces the Pi starvation-induced virulence of this pathogen. Surprisingly, lack of the σ^VreI^ inhibitor, the VreR anti-σ factor, also diminishes the virulence of *P. aeruginosa*. By transcriptomic analyses we show that VreR modulates gene expression not only in a σ^VreI^-dependent but also in a σ^VreI^-independent manner. This includes potential virulence determinants and transcriptional regulators that could be responsible for the reduced virulence of the ΔvreR mutant.

## Introduction

The pathogen *Pseudomonas aeruginosa* produces several life-threatening infections in humans, especially in immunocompromised, cancer, burn and cystic fibrosis patients and it is one of the primary reasons of hospital-acquired infections^1–4^. Importantly, antibiotic resistance among this pathogen has escalated globally over the past three decades and several outbreaks in hospitals have highlighted the need of controlling multi-drug resistant *P. aeruginosa* infection and spread^5^. Indeed, the World Health Organization has declared this bacterium the second priority pathogen demanding research and development of new treatment strategies. Therefore, there is an enormous research need to identify new molecular targets that permit the inhibition or elimination of this pathogen.

*P. aeruginosa* is highly metabolic versatile and harbors multiple virulence factors that enable this pathogen to infect essentially any mammalian tissue^3,6^. Central to the infectious process is the ability of the pathogen to adapt to changing environments and *P. aeruginosa* produces many global regulators and signal transduction systems that facilitate its adaptation^7,8^. Regulation of gene expression in bacteria occurs initially at the transcription initiation level through the modulation of the affinity of the RNA polymerase (RNAP) for the DNA. Such affinity can be modified through the replacement of the sigma (σ) subunit of the RNAP, which is the subunit responsible of promoter recognition and thus of the specificity of the RNAP, and/or by transcriptional regulators that enhance or repress RNAP binding and activity^9^. *P. aeruginosa* contains a plethora of these regulatory proteins, which often function in response to specific cues. Among them, sigma factors of the extracytoplasmic function sigma (σ^ECF^) factor family are important signal-responsive regulatory proteins in *P. aeruginosa*^10,11^. The σ^ECF^-mediated signaling in this bacterium generally involves the function of an anti-σ factor^10,11^. Most *P. aeruginosa* anti-σ factors are single-pass transmembrane proteins that contain a cytosolic N-terminal domain that binds the σ^ECF^ factor and occludes the RNAP binding determinants, and a periplasmic C-terminal domain required for signal transduction. In response to a specific inducing signal, the anti-σ factor usually undergoes regulated proteolysis^12–15^, which leads to the release of an active σ^ECF^ factor that binds to the RNAP and promotes transcription of the signal response genes.

*P. aeruginosa* contains between 19 and 21 σ^ECF^ factors that cluster into nine different phylogenetic groups^10^. Most belong to the iron starvation (IS) group and are expressed in iron limiting conditions together with an anti-σ factor. Post-translational activation of IS σ^ECF^ factors often occurs in response to the presence of an iron chelating compound (i.e. siderophores, heme/hemoglobin, iron-citrate) by a signal transduction cascade known as cell-surface signaling (CSS) that also involves an outer membrane-located TonB-dependent transducer (TBDT)^10,16,17^. IS σ^ECF^ factors promote transcription of iron acquisition functions and regulate iron homeostasis, which are essential processes for *P. aeruginosa* to spread and colonize the host. Besides, several *P. aeruginosa* IS σ^ECF^ factors stimulate the transcription of virulence determinants^10,11,16^. The second most abundant σ^ECF^ group in *P. aeruginosa* is formed by the RpoE-like σ^ECF^ factors. These σ factors are activated in response to cell envelope stress and trigger expression of functions that mitigate stress and maintain the integrity of the bacterial cell envelope, thus ensuring pathogen survival^10,11^. While required during infection to cope with stresses produced by the host immune response (e.g. increased temperature, formation of oxygen reactive species or osmotic changes), *P. aeruginosa* stress-responsive σ^ECF^ factors also promote expression of important virulence determinants (i.e. the exopolysaccharide alginate)^10,11^. The signaling cascade activating these σ^ECF^ factors usually involves an anti-σ factor but not an outer membrane TBDT^10,11^.

**Figure 1:**
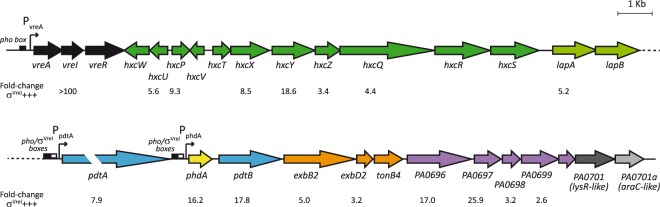
Genetic organization of the σ^VreI^ regulon. Transcriptional organization of the *vreAIR* locus (black) and the downstream σ^VreI^-regulated genes (colored). Block arrows represent the different genes, their relative sizes, and their transcriptional orientation, with the name of the gene or the PA number (http://www.pseudomonas.com/) indicated below the arrow. The promoters and regulatory boxes identified within this locus are indicated^19,20,62^. Numbers indicate the fold-change in the expression of the gene in cells overproducing σ^VreI^ as determined earlier by microarray^21^. The *hxc* genes (dark green) encode a type II secretion system involved in the secretion of the low molecular weight alkaline phosphatase LapA (light green)^62^. *pdtA* and *pdtB* (blue) encode a functional two-partner secretion (TPS) system involved in *P. aeruginosa* virulence in the *C. elegans* model^63^. *phdA* (yellow) encodes a homologue of the prevent-host-death (Phd) protein family and is required for biofilm formation and eDNA release^64^. *exbB2-exbD2-tonB4* genes (orange) encode a still uncharacterized putative TonB system. The function of the PA0696-PA0700 gene products (purple) is still unknown. PA0701 (dark grey) encodes a putative LysR-like transcriptional regulator and PA0701a (light grey), which is not annotated in the PAO1 genome but it is in the *P. aeruginosa* PA14 genome, encodes a putative AraC-like transcriptional regulator.

The *P. aeruginosa* σ^VreI^ factor was initially classified within the IS group^18^. However, our recent analyses showed that expression of this σ factor is not regulated by iron, but by inorganic phosphate (Pi)^19,20^. This was in agreement with our initial observations showing that σ^VreI^ does not promote expression of iron acquisition functions^21^. σ^VreI^ is encoded by the *vreAIR* operon together with a CSS-like receptor protein (VreA) and a transmembrane anti-σ factor (VreR)^19,21^. While the anti-σ role of VreR has been demonstrated^19^, the function of VreA in the σ^VreI^ signaling cascade, if any, is at present unknown. The N-terminal domain of VreA resembles that of CSS receptors^21^, which is the domain that interacts with the anti-σ factor upon signal recognition triggering activation of the CSS cascade and the IS σ^ECF^ factor^16^. However, VreA lacks the C-terminal β-barrel domain of CSS receptors, which is the domain required for the uptake of the CSS ligand (i.e. siderophore, heme)^21^. We initially hypothesized that VreA could be involved in signaling but not in transport^21^; however, our recent analyses showed that, *in vitro*, VreA is not required for σ^VreI^ activation^19^. Transcription of the *vreAIR* operon takes place in Pi limited conditions and requires the phosphate transcriptional regulator PhoB^19^. Besides promoting *vreI* transcription, PhoB is also required for transcription of the σ^VreI^ regulon genes by recruiting the σ^VreI^-RNAP complex to the promoter region of these genes^19^. Our earlier microarray analyses showed that the σ^VreI^ regulon includes several *P. aeruginosa* virulence determinants (Fig. 1)^21^. In accordance, constitutive activation of σ^VreI^ increases *P. aeruginosa* pathogenicity^21^. Antibodies directed against secreted proteins of the σ^VreI^ regulon (i.e. PdtA, Fig. 1) are detected in the serum of *P. aeruginosa* infected patients^21^. Moreover, interaction of *P. aeruginosa* with host cells promotes transcription of σ^VreI^ regulon genes^22,23^. Together this suggests that transcription of the σ^VreI^ regulon occurs during infection and thus that σ^VreI^ is active in this condition. However, there is no direct proof yet demonstrating the activation of σ^VreI^ *in vivo*.

The aim of this work was to investigate whether the σ^VreI^ factor is active during infection. Moreover, because *vreI* is expressed under Pi starvation, a condition often encountered by pathogens in the host environment that is known to induce a virulent phenotype in *P. aeruginosa*^24^, we also aimed at determining the contribution of this σ factor to the Pi starvation-induced virulence of this pathogen. Using zebrafish embryos and a human respiratory epithelial cell line as *P. aeruginosa* hosts we show for the first time that σ^VreI^ is indeed activated during infection and that lack of the σ^VreI^/VreR signaling proteins diminishes the Pi starvation-induced virulence of this pathogen.

## Results

### σ^VreI^ and VreR are required for *P. aeruginosa* virulence in zebrafish embryos

**Figure 2:**
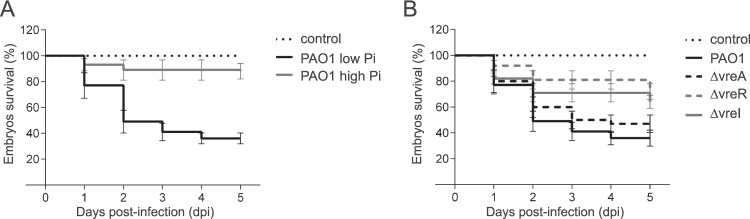
Survival of zebrafish embryos upon infection with *P. aeruginosa*. One day post-fertilization embryos were injected with ∼1000 CFU of the *P. aeruginosa* PAO1 wild-type strain grown either in Pi-restricted or Pi-sufficient conditions (**A**) or with the indicated PAO1 isogenic mutant grown under Pi starvation (**B**). Uninfected control (non-injected) is shown. Data represents the mean ± SD of four biologically independent replicates (N = 4) with 20 embryos/group in each replicate. P-values were calculated by log-rank (Mantel-Cox) test.

The virulence of *P. aeruginosa* was assayed using zebrafish (*Danio rerio*) embryos as host. *P. aeruginosa* is able to lethally infect zebrafish embryos when the number of bacterial cells injected exceeds the phagocytic capacity of the embryo^21,25,26^. Because the expression of the *vreAIR* operon is induced under phosphate (Pi) starvation^19,20^, we first determined the effect of the Pi concentration in *P. aeruginosa* virulence. Thus, *P. aeruginosa* cells previously grown either in low or high Pi conditions were injected into the blood stream of one-day post-fertilization embryos to generate a systemic infection and embryo survival was monitored during five days. Survival of the embryos injected with PAO1 wild-type cells grown in low Pi was considerably lower than that of the embryos injected with PAO1 cells grown in high Pi conditions (P < 0.001) (Fig. 2A), which indicates that Pi starvation induces a *P. aeruginosa* virulent phenotype. The contribution of σ^VreI^ to this phenotype was determined using a null ΔvreI mutant. The virulence of this mutant was significantly lower than that of the PAO1 wild-type strain (P < 0.05) (Fig. 2B), which suggests that σ^VreI^ contributes to the low Pi-induced virulence of *P. aeruginosa*. Unexpectedly, a ∆vreR anti-σ factor mutant in which σ^VreI^ is highly active^19^ also showed attenuated virulence (P < 0.001) (Fig. 2B). This prompts that VreR may have more functions in *P. aeruginosa* than solely controlling σ^VreI^ activity or alternatively, that the timing and/or quantity of the σ^VreI^ response is crucial. In contrast, the absence of the CSS-like receptor protein VreA did not have any effect on *P. aeruginosa* virulence, which was similar for the ΔvreA mutant and the PAO1 strain (P = 0.35) (Fig. 2B). This suggests that this protein is not involved in the σ^VreI^/VreR-mediated virulence of this bacterium.

### σ^VreI^ and VreR are required for *P. aeruginosa* cytotoxicity

**Figure 3:**
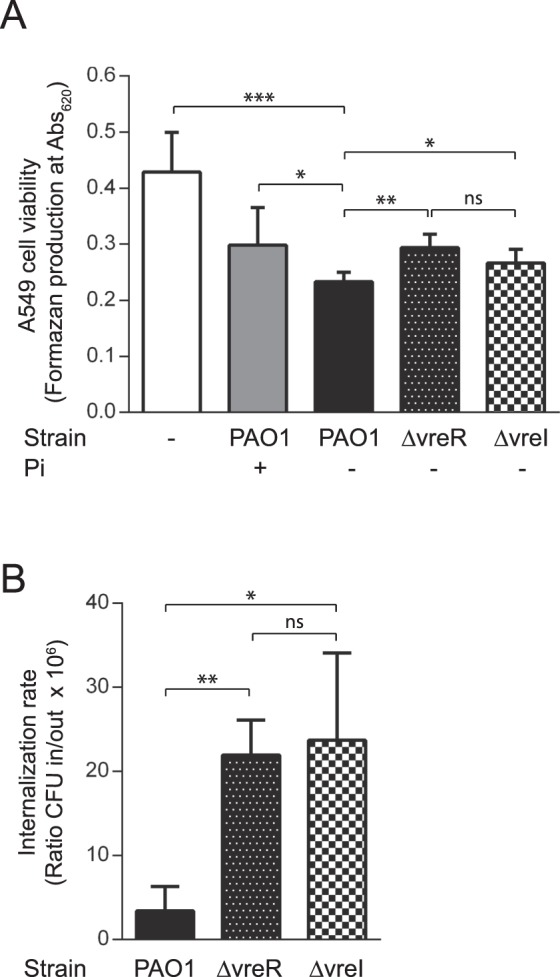
*P. aeruginosa* infections in the A549 cell line. (**A**) A549 cell viability. The *P. aeruginosa* PAO1 wild-type strain and the indicated isogenic mutant were grown in Pi-restricted (−) or Pi-sufficient (+) conditions prior to infection. Formazan production upon addition of the MTT tretazolium salt was determined spectrophotometrically at 620 nm. Uninfected cells (white bar) were used as control. (**B**) *P. aeruginosa* internalization into A549 cells. A549 cells were infected with the indicated *P. aeruginosa* strain previously grown in Pi-restricted conditions. Internalization is reported as the ratio between bacteria CFU inside (in) the A549 cells and CFU in the culture supernatant (out). In both panels data are means ± SD from three biological replicates (N = 3). P-values were calculated by unpaired two-tailed *t*-test as described in Materials and Methods and brackets indicate the comparison to which the P-value applies.

*P. aeruginosa* commonly affects the respiratory tract in humans. Therefore, we used the A549 human respiratory epithelial cell line as *P. aeruginosa* host. First we assayed the cytotoxicity of *P. aeruginosa* against the eukaryotic cells by determining A549 cell viability after co-incubation with the bacteria. Similar to the results obtained in the zebrafish embryos, we observed that growth of *P. aeruginosa* under Pi starvation slightly increases the bacterial cytotoxicity as the eukaryotic cells were more damaged by bacteria grown in low rather than in high Pi medium (Fig. 3A). Both σ^VreI^ and VreR contribute to this phenotype, because mutants lacking these proteins were significantly less efficient in damaging the A549 cells than the PAO1 wild-type strain (Fig. 3A).

We also measured internalization of *P. aeruginosa* into A549 cells. Although considered an extracellular pathogen, *P. aeruginosa* is able to enter into non-phagocytic host cells such as epithelial cells^27,28^. The internalization efficiency of a given strain depends on several factors including the T3SS profile^29,30^. Strains that are more efficient in internalizing are less cytotoxic while less invasive strains kill the eukaryotic cells more rapidly. Therefore, there is an inverse correlation between internalization and cytotoxicity, and thus between internalization and acute virulence^31^. Upon *P. aeruginosa* infection of A549 cells, we observed a ∼3-fold increase in the internalization of the ∆vreI and ∆vreR mutants as compared to the PAO1 wild-type strain (Fig. 3B). This is consistent with the lower cytotoxicity displayed by these mutants (Fig. 3A) and with the reduced capacity of these mutants to produce a systemic infection in zebrafish embryos (Fig. 2B).

### VreR influences *P. aeruginosa* gene expression also in a σ^VreI^-independent manner

The phenotype of the ΔvreR mutant, in which reduced instead of increased virulence was observed, prompted us to analyze gene expression in this mutant by RNA-seq. We compared the transcriptome of the *P. aeruginosa* PAO1 wild-type strain with that of the ΔvreR mutant upon bacterial growth in Pi starvation, a condition known to induce *vreR* expression^19^. A total of forty-four transcripts were more abundant in the ΔvreR mutant than in the PAO1 wild-type strain, and nine were less abundant (including the *vreR* transcript) (Table 1). Increased expression of some of these genes in the ΔvreR mutant was confirmed by qRT-PCR (Fig. S1). Of the forty-four upregulated genes, nineteen are located immediately downstream the *vreAIR* locus and most of them belong to the σ^VreI^ regulon previously identified by microarray of cells overproducing σ^VreI^ (Fig. 1)^21^. Other upregulated transcripts belong to genes located in different loci in the *P. aeruginosa* PAO1 genome (Table 1). These include genes encoding metabolic and energy obtaining functions, transport, and several regulators of gene expression (Table 1).

**Table 1:** Differentially expressed *P. aeruginosa* genes in ΔvreR versus PAO1^a^.

ORF	Gene name	Function and reference^b^	log2(fold-change)^c^	test stat^d^	q-value
PA0141	—	Polyphosphate kinase 2 (PPK2) (utilizes poly P to make GTP, which is needed for the synthesis of alginate)^65^	1,22	4.39	0.00587
PA0200	—	Unknown function	2.56	8.52	0.00587
PA0677	*hxcW*	Hxc T2SS^62^	4.26	8.46	0.00587
PA0680	*hxcV*	Hxc T2SS^62^	2.92	9.54	0.00587
PA0681	*hxcT*	Hxc T2SS^62^	3.99	12.30	0.00587
PA0682	*hxcX*	Hxc T2SS^62^	4.15	11.79	0.00587
PA0683	*hxcY*	Hxc T2SS^62^	4.69	5.17	0.00587
PA0685	*hxcQ*	Hxc T2SS^62^	3.75	4.76	0.01063
PA0688	*lapA*	Low-molecular-weight alkaline phosphatase A, secreted by the Hxc T2SS^62^	5.77	12.44	0.00587
PA0690	*pdtA*	TPS partner A, large secreted exoprotein^63^	HIDATA^e^	—	—
PA0691	*phdA*	Prevent-host-death protein A, involved in biofilm formation^64^	4.16	9.01	0.00587
PA0692	*pdtB*	TPS partner B, outer membrane protein involved in *pdtA* secretion^63^	4.67	10.31	0.00587
PA0693	*exbB2*	ExbB proton channel	4.31	9.50	0.00587
PA0694	*exbD2*	ExbD protein family	4.46	13.55	0.00587
PA0695	*tonB4*	TonB energy protein	4.73	11.70	0.00587
PA0696	—	Unknown function, putative outer membrane porin	4.22	9.71	0.00587
PA0697	—	Unknown function, structural homology with channel-forming colicins	4.98	12.94	0.00587
PA0698	—	Putative sensory transduction regulator of the YbjN protein family	4.91	13.18	0.00587
PA0699	—	Probable peptidyl-prolyl cis-trans isomerase, PpiC-type	4.59	9.91	0.00587
PA0701	—	Probable LysR-type transcriptional regulator	4.01	10.74	0.00587
PA0701a	—	Probable AraC-type transcriptional regulator	2.05	5.44	0.00587
PA1196	*ddaR*	σ^54^ dependent transcriptional regulator, regulates methylarginine degradation^66^	1.23	4.32	0.01063
PA1414	—	Unknown function	1.90	4.60	0.01063
PA1429	—	Probable cation-transporting P-type ATPase	1.53	4.30	0.00587
PA1546	*hemN*	Oxygen-independent coproporphyrinogen-III oxidase (heme biosynthesis)^67^	2.19	5.12	0.00587
PA1556	*ccoO2*	Cytochrome c oxidase, cbb3-type, CcoO subunit (energy metabolism, electron transport)	2.40	8.24	0.00587
PA1673	—	Probable bacteriohemerythrin (non-heme diiron oxygen transport proteins)	2.43	8.09	0.00587
PA1746	—	Unknown function. Orthologue to Appr-1-p (ADP-ribose-1”-monophosphate) processing protein	1,81	5.95	0.00587
PA2753	—	Unknown function	1.42	5.09	0.00587
PA3278	—	Unknown function	1.26	4.31	0.00587
PA3305.1	*phrS*	Non-coding RNA involved in quorum sensing regulation^68^	2.79	5.94	0.00587
PA3337	*rfaD*	ADP-L-glycero-D-manno-heptose-6-epimerase	2.38	7.70	0.00587
PA3458	—	Probable transcriptional regulator of the MarR family	1.18	3.99	0.00587
PA3880	—	Unknown function	2.04	6.30	0.00587
PA4067	*oprG*	Outer membrane porin, transport of cations and small aminoacids^69^	2.89	6.99	0.00587
PA4159	*fepB*	Ferrienterobactin-binding periplasmic protein^70^	1.45	5.13	0.00587
PA4348	—	Unknown function, contains a metallo-beta-lactamase domain	3.047	9.90	0.00587
PA4358	*feoB*	Fe^2+^ transporter^71^	2.51	6.73	0.00587
PA4359	*feoA*	Fe^2+^ transporter, probable activator of FeoB^71^	3.57	9.92	0.00587
PA4577	—	Unknown function, probable TraR/DksA family transcriptional regulator	1.58	5.40	0.00587
PA4610	—	Unknown function, probable copper export protein	1.26	4.48	0.01063
PA5027	*dadA*	Universal stress (UspA)-like protein	2.35	6.25	0.00587
PA5427	*adhA*	Alcohol dehydrogenase	1.95	5.12	0.00587
PA5475	—	Unknown function, N-acetyltransferase domain	2.81	7.02	0.00587
PA0676	*vreR*	Anti-σ factor^19^	−9.79	−19.48	0.00587
PA0878		Unknown function	−1.30	−4.21	0.00587
PA2356	*msuD*	Methanesulfonate sulfonatase (sulfur metabolism)^72^	−1.82	−4.58	0.00587
PA2357	*msuE*	NADH-dependent FMN reductase (sulfur metabolism)^72^	−2.05	−6.08	0.00587
PA3510	—	Unknown function	−1.10	−3.85	0.01063
PA4022	*hdhA*	Hydrazone dehydrogenase^73^	−1.01	−3.57	0.04697
PA4280.2		23 S rRNA^74^	−3.89	−7.68	0.00587
PA4690.5		16 S rRNA^74^	−2.08	−5.26	0.00587
PA4690.2		23 S rRNA^74^	−2.08	−5.54	0.00587

^a^Significant differentially expressed genes were obtained by Cufflinks 2.2.1 analyses ref. ^60^.

^b^T2SS, type II secretion system; TPS, two partner secretion; rRNA, ribosomal RNA; tRNA, transfer RNA.

^c^The (base 2) log of the fold change (FPKM-ΔvreR/FPKM-PAO1 being FPKM fragments per kilobase per million fragments mapped).

^d^Value of the test statistic used to compute significance of the observed change in FPKM ref. ^60^.

^e^Too many fragments of the *pdtA* mRNA in the ΔvreR sample.

**Figure 4:**
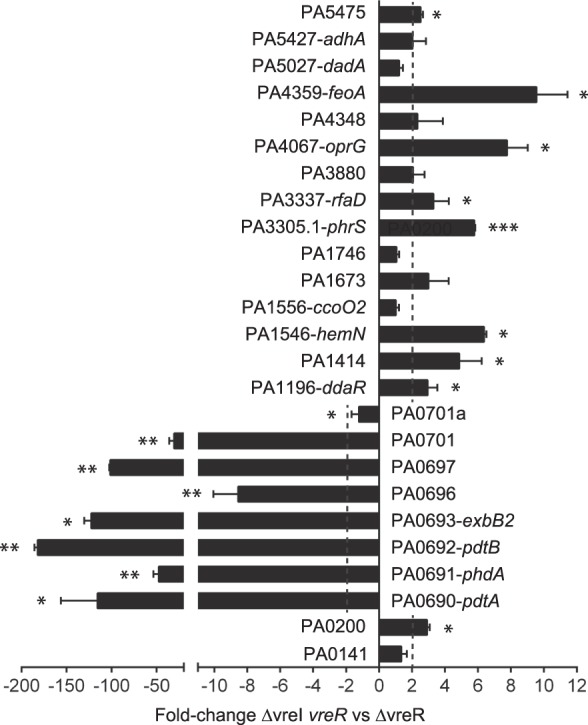
Differential gene expression in the *P. aeruginosa* ΔvreR and ΔvreI *vreR* mutants. mRNA levels of the indicated genes were obtained by qRT-PCR upon growth of the *P. aeruginosa* mutants in low Pi medium. The 2^−ΔΔCT^ method was used to determine the fold-change range in gene expression in ΔvreI *vreR* versus ΔvreR. Data are means ± SD from three biological replicates (N = 3) each one including three technical replicates. P-values were calculated by one-sample *t*-test to a hypothetical value of 1 as described in Materials and Methods.

We then wondered whether the increased expression of these genes in the ΔvreR mutant was due to σ^VreI^, which is highly abundant and active in this mutant^19^. Therefore, we compared the relative expression of these genes in the ΔvreR mutant with that of the ΔvreI mutant in which *vreR* is also not produced due to a polar effect of the *vreI* deletion on the expression of the downstream *vreR* gene^19^ (referred as a ΔvreI *vreR* mutant in Fig. 4). A total of twenty-five genes were selected, eight known to belong to the σ^VreI^ regulon (Fig. 1). Indeed, these eight genes were expressed considerably less in the ΔvreI *vreR* mutant than in ΔvreR (Fig. 4). This shows that the higher expression of these genes in the ΔvreR mutant directly depends on σ^VreI^. However, the relative expression of eight genes that were not previously identified as part of the σ^VreI^ regulon (PA0141, *ccoO2*, PA1673, PA1746, PA3880, PA4348, *dadA* and *adhA*) was similar in both mutants (Fig. 4). This indicates that the expression of these genes is not affected by the absence of σ^VreI^ and therefore that this σ factor is not involved in their transcription. Finally, the expression of nine other genes (PA0200, *ddaR*, PA1414, *hemN*, *phrS*, *rfaD*, *oprG*, *feoA* and PA5475) was higher in the ΔvreI *vreR* mutant than in ΔvreR (Fig. 4). This suggests that σ^VreI^ not only does not mediate the transcription of these genes, but also that presence of this σ factor impairs their expression. Overall, this analysis suggests that VreR has a broader role in gene regulation beyond its direct involvement in the σ^VreI^ signaling pathway.

### σ^VreI^ is activated *in vivo* during infection

**Figure 5:**
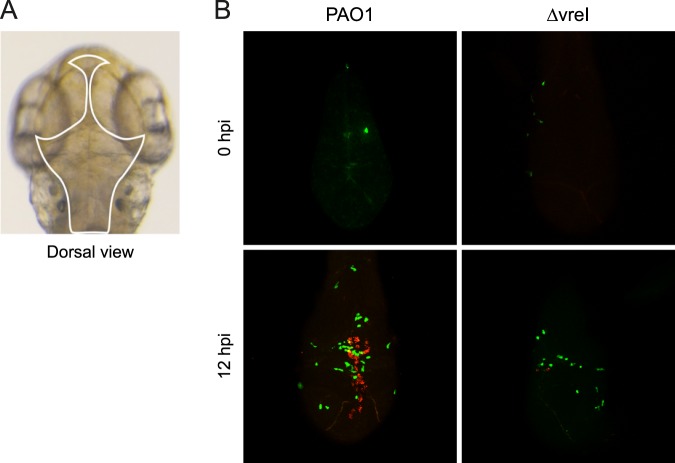
σ^VreI^ activation during *P. aeruginosa* infection in zebrafish embryos. (**A**) Dorsal view of the head of a two days post-fertilization zebrafish embryo. The hindbrain ventricle where *P. aeruginosa* was injected is highlighted. (**B**) Confocal images of the head of embryos injected in the hindbrain ventricle with ∼2000 CFU of the *P. aeruginosa* PAO1 wild-type strain or its isogenic ΔvreI mutant bearing the σ^VreI^-dependent *pdtA::rfp* transcriptional fusion (pMP0690mCherry plasmid, Table S1) (red channel) at 0 and 12 hpi. *P. aeruginosa* was grown in high Pi medium prior injection. Neutrophils expressing constitutively a green fluorescence protein (gfp) are also visualized (green channel). Images are representative of three independent experiments (N = 3).

To analyze whether σ^VreI^ is activated *in vivo*, we used zebrafish embryos and A549 cells as hosts and a σ^VreI^-dependent fluorescent construct in which the promoter of the σ^VreI^ regulated gene *pdtA* (Fig. 1) was cloned in front of a red fluorescent protein (*rfp)* gene (Table S1). *P. aeruginosa* PAO1 and ΔvreI strains were grown in high Pi conditions to avoid σ^VreI^ expression and activation prior to infection^19^. Bacteria were injected in the hindbrain ventricle of the zebrafish embryos (Fig. 5A) to produce an infection that remains initially localized, which facilitates fluorescence measurements. Directly after injection with either strain [0 hours post-infection (hpi)], red fluorescence was undetectable, indicating that there was no transcription from the σ^VreI^-dependent *pdtA* promoter and thus that σ^VreI^ is not active (Fig. 5B, red channel). At this early infection point there was no neutrophil recruitment observed inside the hindbrain ventricle (Fig. 5B, green channel). However, at 12 hpi embryos injected with the PAO1 wild-type strain or the ΔvreI mutant showed recruitment of neutrophils in the head, indicative of an ongoing infection (Fig. 5B, green channel). Importantly, at this time point red fluorescence in bacterial aggregates was observed in the embryos infected with the wild-type but not with the ∆vreI mutant (Fig. 5B, red channel). This shows that *pdtA* expression occurs during infection in a σ^VreI^-dependent manner and therefore suggests that this σ factor is expressed and active in these conditions.

**Figure 6:**
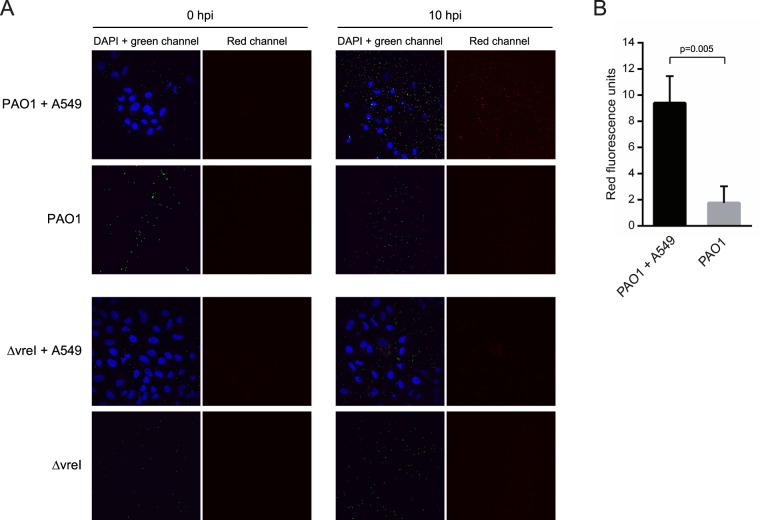
Activation of σ^VreI^ upon interaction of *P. aeruginosa* with A549 eukaryotic cells. (**A**) Confocal images of *P. aeruginosa*-A549 co-cultures at 0 and 10 hpi. *P. aeruginosa* PAO1 wild-type strain and its isogenic ∆vreI mutant expressing a green fluorescent protein (gfp) constitutively (from the pBBRmEos3.1 plasmid, Table S1) (green channel) and containing the σ^VreI^-dependent *pdtA::rfp* transcriptional fusion (pMP0690mCherry plasmid, Table S1) (red channel) were grown in high Pi medium and inoculated in A549-containing and A549-free cultures. A549 cells DNA was stained with DAPI (blue channel). Images are representative of three independent experiments (N = 3). (**B)** Quantification of the red fluorescence intensity observed in (A) was performed as described in Materials and Methods, and the total corrected cellular fluorescence (TCCF) is given. Data are means ± SD from three biological replicates (N = 3). P-value was calculated by unpaired two-tailed *t*-test.

**Figure 7:**
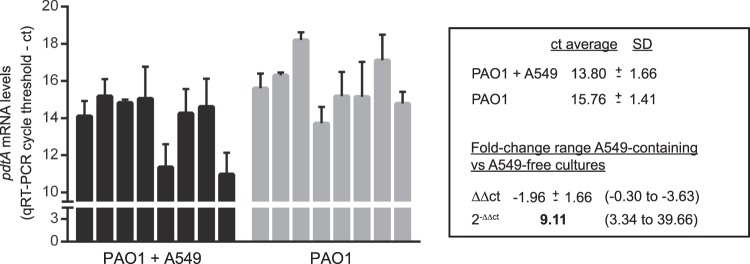
*pdtA* mRNA levels upon *P. aeruginosa* interaction with A549 cells. The *P. aeruginosa* PAO1 wild-type strain was grown in high Pi medium and inoculated in A549-containing and A549-free cultures. At 3 hpi, total RNA was extracted and *pdtA* mRNA levels determined by qRT-PCR. Data plotted are the result of eight biologically independent replicates, each bar representing means ± SD of the three technical replicates performed on each biological replicate. The cycle threshold (ct) average and the standard deviation (SD) of each condition is indicated. The 2^−ΔΔCT^ method was used to determine the fold-change range in *pdtA* expression in A549-containing versus A549-free cultures. The fold-change range taking into account the SD is shown between brackets.

We also analyzed σ^VreI^ activation upon interaction of *P. aeruginosa* with the A549 human lung epithelial cell line. Green fluorescence-labelled *P. aeruginosa* PAO1 and ΔvreI strains bearing the σ^VreI^-dependent *pdtA::rfp* transcriptional fusion were inoculated in A549 cultures. As control, bacteria were also inoculated in cell-free cultures. At 0 hpi, red fluorescence in either extracellular or internalized *P. aeruginosa* cells was undetectable (Fig. 6A). However, at 10 hpi red fluorescence was observed in the PAO1 wild-type cells and was considerably higher in the A549-containing cultures than in the A549-free cultures (P < 0.01) (Fig. 6A,B). In contrast, fluorescence remained undetectable in the ΔvreI mutant independently of the presence or absence of the A549 epithelial cells (Fig. 6A). Together this suggests that the expression of *pdtA* is induced by the presence of the human cells and that this induction depends on σ^VreI^. To confirm this result, we measured *pdtA* expression by qRT-PCR after PAO1 inoculation in A549-free and A549-containing cultures. The cycle threshold (ct) value of a total of eight biologically independent co-incubations was determined (Fig. 7). In A549-containing cultures the expression of *pdtA* increased between 3- and 40-fold compared to the cell-free cultures (Fig. 7). This confirms that expression of *pdtA* is induced upon contact of *P. aeruginosa* with the eukaryotic cell. Altogether, these results indicate that σ^VreI^ is active during infection.

## Discussion

Pi starvation has been described as an important signal for pathogens and indeed Pi amounts are partially linked to the immune status of the host. During major surgical operations and also in patients with severe burns, the reabsorption of Pi by the kidneys is reduced and exudative losses are higher than normal leading to hypophosphatemia, which can intensify upon treatments with bisphosphonates or antivirals^32–34^. During respiratory alkalosis caused by sepsis or mechanical respiration, a redistribution of the Pi into cells occurs and this reduces the extracellular content of Pi^35^. Increased levels of circulating catecholamines, which occurs in CF patients^36^, has been also associated with hypophosphatemia^35^. Evolution has benefited pathogens that are able to recognize and react to this marker^37^, thus exploiting the lower activity of the host immune system in this condition^38^. *P. aeruginosa* recognizes low Pi environments through the phosphate-specific ABC transport Pst system, which in Pi limiting conditions transports phosphate and triggers activation of the PhoR/PhoB two-component system^37^. Activated PhoR histidine kinase phosphorylates the transcriptional response regulator PhoB, which in turn promotes expression of the so-called *pho* regulon that in *P. aeruginosa* comprises several virulence factors^37^. In accordance, Pi starvation enhances *P. aeruginosa* virulence in zebrafish embryos (Fig. 2A), as also observed in mice and nematodes^24,39–41^, whereas excess of Pi reduces virulence (Fig. 2A). The *pho* regulon includes the *vreAIR* operon and the σ^VreI^ regulon (Fig. 1)^19^, and we have shown here that ΔvreI and ΔvreR mutations impair the low Pi-induced *P. aeruginosa* virulence (Figs. 2B and 3). Although the attenuated virulence of the ΔvreI mutant could be due to the lack of *vreR* expression in this mutant^19^, its phenotype is in line with previous results showing that overproduction of σ^VreI^ considerably increases *P. aeruginosa* pathogenicity^21^. In fact, it was surprising to find that the absence of the anti-σ factor VreR also led to attenuated virulence, despite the fact that σ^VreI^ is considerably active in the ΔvreR mutant^19^. Our transcriptomic and qRT-PCR analyses have revealed that VreR influences gene expression not only in a σ^VreI^-dependent but also in a σ^VreI^-independent manner. This includes several functions that could affect *P. aeruginosa* virulence (e.g. the PA0141/PPK2 required for alginate synthesis, the non-coding *phrS* RNA involved in quorum sensing regulation, the universal stress response *dadA*), and several regulators of gene expression (e.g. *ddaR*, PA3458/MarR-like regulator, PA4577/DksA-like regulator) (Table 1). How VreR modulates gene expression independently of σ^VreI^ is at present unknown but likely involves some of these regulatory proteins. In contrast, the third component of the operon, VreA, does not seem to play a role in the low Pi-induced virulence of *P. aeruginosa*. In fact, VreA does not seem to be involved in σ^VreI^ activation either *in vitro*^19^ or *in vivo* (Fig. 2B), which suggests that the σ^VreI^/VreR signaling cascade does not require this protein.

Furthermore, we have shown in this work that σ^VreI^ is active *in vivo* during infection. *pdtA* expression, which *in vitro* completely depends on the σ^VreI^/VreR signaling cascade and the PhoB regulator^19^, occurs during *P. aeruginosa* infections in zebrafish embryos and upon interaction with human epithelial cells, and in these *in vivo* situations expression of *pdtA* also completely depends on σ^VreI^. The activation of σ^VreI^ *in vivo* is in accordance with the increased expression of the *vreAIR* operon and the σ^VreI^ regulon (including *pdtA*) in mouse models of acute and chronic infections^42^, and with the presence of antibodies against the PdtA protein in the serum of *P. aeruginosa* infected patients^21^. Presence of an active σ^VreI^ protein requires Pi limitation conditions in order for *vreI* to be expressed. However, the σ^VreI^ inhibitor, the VreR anti-σ factor, is also expressed in this condition^19^. A basal activity of σ^VreI^ is observed under Pi limitation; however, maximal activity of this σ factor requires the removal of VreR^19^. Because activation of *P. aeruginosa* σ^ECF^ factors in response to the inducing signal usually occurs through the regulated proteolysis of the anti-σ factor^12–15^, another signal is expected to be required for complete σ^VreI^ activation. The signal could be host-derived, as described for the σ^V^ factor of the opportunistic pathogens *Clostridioides difficile* and *Enterococcus faecalis*, which is activated by lysozyme, an important component of the innate immune system of many organisms^43^. Alternatively, the activating signal could be produced by *P. aeruginosa* itself in response to the host environment. An example of such a mechanism is the activation of σ^PvdS^ in *P. aeruginosa*, which in response to the iron starvation conditions encountered in the host^44,45^ produces pyoverdine that in turn increases σ^PvdS^ activation and *P. aeruginosa* virulence^46,47^. Another example is the activation of the *P. aeruginosa* σ^AlgT^ factor in response to the oxidative stress generated by the oxygen radicals produced by leucocytes or in response to the elevated temperatures that are often produced in infected hosts^48,49^. Both situations lead to cell envelope stress and the accumulation of misfolded proteins in the bacterial periplasm, which trigger σ^AlgT^ activation^50^. Further research will be conducted to clarify how activation of σ^VreI^ in response to the host occurs.

## Methods

### Bacterial strains and growth conditions

Strains used are listed in Table S1. Bacteria were routinely grown in Luria-Bertani (LB) medium^51^ at 37 °C in a rotary shaker at 200 rpm. For differential expression of the *vreAIR* operon low and high phosphate media were used^19^. When necessary, antibiotics (Sigma-Aldrich) were used at the following final concentrations (μg ml^−1^): ampicillin (Ap), 100; gentamicin (Gm), 20; hygromycin B (Hg), 100; nalidixic (Nal), 10; and tetracycline (Tc), 20.

### Plasmids construction and molecular biology

Plasmids used are described in Table S1 and primers listed in Table S2 and S3. PCR amplifications were performed using Phusion^®^ Hot Start High-Fidelity DNA Polymerase (Finnzymes). All constructs were confirmed by DNA sequencing and transferred to *P. aeruginosa* by electroporation^52^.

### Zebrafish maintenance, embryo care and infection procedure

Transparent adult *casper* mutant zebrafish *(mitfa*^*w2/w2*^*; roy*^*a9/a9*^*)* and adult labelled *Tg(mpx:GFP)*^*i114*^ *casper* zebrafish producing green fluorescent neutrophils^53,54^ were maintained at 26 °C in aerated 5 L tanks with a 10/14 h dark/light cycle. Zebrafish embryos were collected during the first hour post-fertilization (hpf) and kept at 28 °C in E3 medium (5.0 mM NaCl, 0.17 mM KCl, 0.33 mM CaCl·2H_2_O, 0.33 mM MgCl_2_·6H_2_O) supplemented with 0.3 mg/L methylene blue. Prior to infection, 1 or 2 days post-fertilization (dpf) embryos were mechanically dechorionated and anaesthetized in 0.02% (w*/*v) buffered 3-aminobenzoic acid methyl ester (pH 7.0) (Tricaine, Sigma-Aldrich). Zebrafish embryos were individually infected by microinjection with 1 nl of *P. aeruginosa* either in the hindbrain ventricle (localized infection) or in the caudal vein (systemic infection) as described elsewhere^26,55^. All procedures involving zebrafish embryos were according to local animal welfare regulations and in accordance with the EU Animal Protection Directive 86/609 EEC.

### Virulence assay in infected zebrafish embryos

Zebrafish embryos were injected in the caudal vein with ∼1000 colony forming units (CFU) of exponentially grown *P. aeruginosa* cells in low or high phosphate conditions previously resuspended in phosphate-free physiological salt containing 0.5% (w/v) of phenol red. After infection, embryos were kept in 6-well plates containing 60 μg/mL of Sea salts (Sigma-Aldrich) at 32 °C with 20 individually injected embryos in each group per well. Embryo survival was determined by monitoring live and dead embryos at fixed time points during five days. Four biologically independent experiments were performed and the data given are the average. P-values were calculated by log-rank (Mantel-Cox) test.

### Confocal fluorescence imaging of zebrafish embryos

For confocal imaging, zebrafish embryos were injected in the hindbrain ventricle with ∼2000 CFU of *P. aeruginosa* cells containing a σ^VreI^-dependent red fluorescence transcriptional fusion. At 0 and 12 hpi, embryos were fixated overnight in 4% (v/v) paraformaldehyde in phosphate buffered saline (PBS). Before imaging, fixated embryos were embedded in 1.5% (w/v) low-melting-point agarose using an open uncoated 8-well microscopy µ-slide (Ibidi®). Confocal images were generated with a Leica TCS SP8 Confocal Microscope. Leica Application Suite X and ImageJ software was used to process the confocal images, specifically for brightness/contrast enhancements as well as for creating merged images.

### Cytotoxicity assay in A549 human lung epithelial cells

*P. aeruginosa* cytotoxicity on A549 cells was assayed using a colorimetric assay that detects the number of metabolically active eukaryotic cells able to cleave the MTT tetrazolium salt (Sigma-Aldrich) to the insoluble formazan dye. The A549 cell line (ATCC^®^ CCL-185™) was maintained in Dulbecco’s Modified Eagle Medium (DMEM) medium supplemented with 10% (v/v) fetal bovine serum (FBS) (Gibco) in a 5% CO_2_ incubator at 37 °C. One day prior to infection, the A549 cells were placed in 96-well plates at a concentration of 4 ×10^4^ cells/well and cultured in phosphate-free DMEM medium (Gibco) with 5% (v/v) FBS. In this condition, cell mitosis does almost not occur. Late exponentially grown *P. aeruginosa* strains in low or high phosphate conditions were then inoculated at a multiplicity of infection (MOI) of 20. At 3 hpi, 30 μl of a 5 mg/ml MTT solution in PBS was added to the wells and the plates were incubated for 2 h. The culture medium was then removed and 100 μl of dimethyl sulfoxide (DMSO) was added to solubilize the formazan. Production of formazan, which directly correlates to the number of viable cells, was quantified using a scanning multi-well spectrophotometer (Infinite® 200 PRO Tecan) at 620 nm.

### Confocal fluorescence imaging of A549 cells

For confocal imaging, 2 ×10^5^ A549 cells were seeded in 24-well plates containing 11 mm round glass coverslips and phosphate-free DMEM medium with 5% (v/v) FBS one day prior to infection. Infections with green fluorescence-labelled *P. aeruginosa* cells containing a σ^VreI^-dependent red fluorescence transcriptional fusion were performed at a MOI of 10. At 0 and 10 hpi, the cells were fixated with 4% (v/v) paraformaldehyde in PBS. Samples were washed with PBS and coverslips were mounted on glass slides containing Fluoroshield mounting medium with DAPI (Sigma-Aldrich) to retain fluorescence and stain the A549 cells DNA. Confocal images were generated with a Nikon A1R confocal scanning laser microscope. NIS-Elements and ImageJ software were used to process the confocal images. Total corrected cellular fluorescence (TCCF) was calculated using the following equation: TCCF = integrated density − (area of selected cell × mean fluorescence of background readings), as described before^56,57^.

### Internalization assay

To enumerate bacteria internalized into A549 cells, a polymyxin B protection assay was performed with *P. aeruginosa* strains grown at late exponential phase in low phosphate conditions. A549 cells were cultured in 24-well plates at a concentration of 2 ×10^5^ cells/well in phosphate-free DMEM medium supplemented with 5% (v/v) FBS. Bacterial infections were performed at a MOI of 10. At 6 hpi, the culture supernatants were collected and serial dilutions plated on LB for bacterial counting. Fresh DMEM medium containing 20 μg/ml polymyxin B was added to the infection wells and incubated during 45 min to kill extracellular bacteria. Afterward, the antibiotic-containing medium was removed and the cells were lysed with 1% (v/v) Triton X-100 (Sigma-Aldrich) in PBS during 10 min. Serial dilution of the lysed cells were plated on LB for bacterial counting. Internalization is reported as the ratio between the CFU after the lysis of the A549 cells and CFU in the culture supernatant.

### RNA isolation

Total RNA was extracted by the hot phenol method using the TRI® Reagent protocol (Ambion) as described elsewhere^58^ and subjected to two DNase I treatments with the Turbo DNA-free kit (Ambion) and RNaseOUT (Invitrogen). RNA quality, including purity, integrity and yield, was assessed by electrophoresis of 1 µg of total RNA and by UV absorption at 260 nm in a ND-1000 spectrophotometer (NanoDrop Technologies, USA).

### Quantitative RT-PCR analyses

A549 cells cultured in 6-well plates in phosphate-free DMEM medium with 5% (v/v) FBS were infected with exponentially grown *P. aeruginosa* cells in high phosphate conditions at a MOI of 10. At 3 hpi, the culture supernatants were spun down at 1600 × g and 4 °C and total RNA was isolated. cDNA from eight biologically independent replicates was produced in triplicate by reverse transcription reactions of 0.5–1 µg RNA using SuperScript II reverse transcriptase (Invitrogen) and random hexamers as primers according to the protocol supplied. Real-time PCR amplifications were carried out on a MyiQ2 system (Bio-Rad) associated with iQ5 optical system software (version 2.1.97.1001) in 11.5 µl reaction mixture containing 6.25 µl of iQ SYBR green Supermix (Bio-Rad), 0.4 µM of each primer (Table S2) and 1 µl of the template cDNA (diluted 1000-fold when measuring the 16S rRNA reference gene). Thermal cycling conditions were the following: one cycle at 95 °C for 10 min and then 40 cycles at 95 °C for 15 s, 65 °C for 30 s, and 72 °C for 20 s, with a single fluorescence measurement per cycle according to the manufacturers’ recommendations. Melting curve analyses were performed by gradually heating the PCR mixture from 55 to 95 °C at a rate of 0.5 °C per 10 s for 80 cycles. The relative expression of the genes was normalized to that of 16 S rRNA and the results were analyzed by means of the comparative cycle threshold (∆∆ct) method^59^.

### RNA-seq analysis

*P. aeruginosa* PAO1 and its isogenic ΔvreR mutant were grown until late exponential phase in low Pi medium and total RNA was isolated. A mixture of the three isolations was used for RNA-seq analyses, which were performed at Era7 Bioinformatics (Granada, Spain). First, the rRNA from 4 µg of total RNA was removed using the Ribo-Zero rRNA removal kit (Illumina) following the manufactures’ recommendations. Sequencing libraries were prepared with the NEBNext Ultra directional RNA library Prep kit (New England Biolabs). RNA was fragmented at 94 °C for 7.5 min and first strand cDNA synthesis was performed at 42 °C during 50 min using the adaptor and primers recommended by the manufacturer (NEBNext Multiplex Oligos, Illumina). Samples were then processed on the Illumina NextSeq. 500 Sequencer in one run with a read length of 2 × 75 bp. The pipeline recommended in the tool Cufflinks 2.2.1^60^ was used to analyze the data and obtain the set of differentially expressed genes following these steps: 1) Raw reads quality analysis with the tool FastQC (http://www.bioinformatics.babraham.ac.uk/projects/fastqc); 2) Reads mapping to the *P. aeruginosa* PAO1 reference genome (NCBI reference sequence NC_002516.2) with the tool Bowtie integrated in the Tophat suite^61^; and 3) analysis of differences in gene expression with Cufflinks 2.2.1^60^. All the samples passed the quality analysis. Tests related to overrepresented sequences failed in some of the samples (corresponding to overexpressed genes in this case, e.g. *pdtA*, Table 1). Significant differentially expressed genes depending on whether P is greater than the false discovery rate (FDR) after Benjamini-Hochberg correction for multiple-testing (as indicated in the Cufflinks 2.2.1 tool) are shown in Table 1. P-value is below 0.0001 in all cases. RNA-seq data set have been deposited in NCBI’s Gene Expression Omnibus (GEO) database under accession number GSE122253 (https://www.ncbi.nlm.nih.gov/geo/query/acc.cgi?acc=GSE122253). RNA-seq results were confirmed by qRT-PCR using RNA from three biological replicates obtained from *P. aeruginosa* cells grown in the same conditions that for the RNA-seq assay and primers listed in Table S2.

### Other bioinformatics analyses

Statistical analyses are based on *t*-test in which two conditions are compared independently. P-values from raw data were calculated by independent two-tailed *t*-test and from ratio data to the control by one-sample *t*-test using GRAPHPAD PRISM version 5.01 for Windows and are represented in the graphs by ns, non-significant; *P < 0.05; **P < 0.01; and ***P < 0.001.

## Supplementary information

Supplementary Information.

## Data Availability

The RNA-seq data set is openly available in NCBI’s Gene Expression Omnibus (GEO) database under accession number GSE122253 (https://www.ncbi.nlm.nih.gov/geo/query/acc.cgi?acc=GSE122253).
