# Supplementary material for: The extracytoplasmic function sigma factor σVreI is active during infection and contributes to phosphate starvation-induced virulence of Pseudomonas aeruginosa
Source: Sci Rep. 2020 Feb 21;10:3139. doi: 10.1038/s41598-020-60197-x (PMC7035377; doi:10.1038/s41598-020-60197-x)
Supplement: Supplementary file 1 — Supplementary Information. [file 41598_2020_60197_MOESM1_ESM.pdf]

Supplementary Information

The extracytoplasmic function sigma factor σ^{VreI} is active during infection and contributes to phosphate starvation-induced virulence of *Pseudomonas aeruginosa*

Joaquín R. Otero-Asman[a], José M. Quesada[a], Kin K. Jim[b], Alain Ocampo-Sosa[c], Cristina Civantos[a], Wilbert Bitter[b], and María A. Llamas[a]*

[a]Department of Environmental Protection, Estación Experimental del Zaidín-Consejo Superior de Investigaciones Científicas, Granada, Spain; [b]Department of Medical Microbiology and Infection Control, Amsterdam University medical centers, location VU University, Amsterdam, The Netherlands; [c]Service of Microbiology, Hospital Universitario Marqués de Valdecilla-Instituto de Investigación Sanitaria Valdecilla, Santander, Spain.

***Correspondence:** Dr. María A. Llamas, email: marian.llamas@eez.csic.es

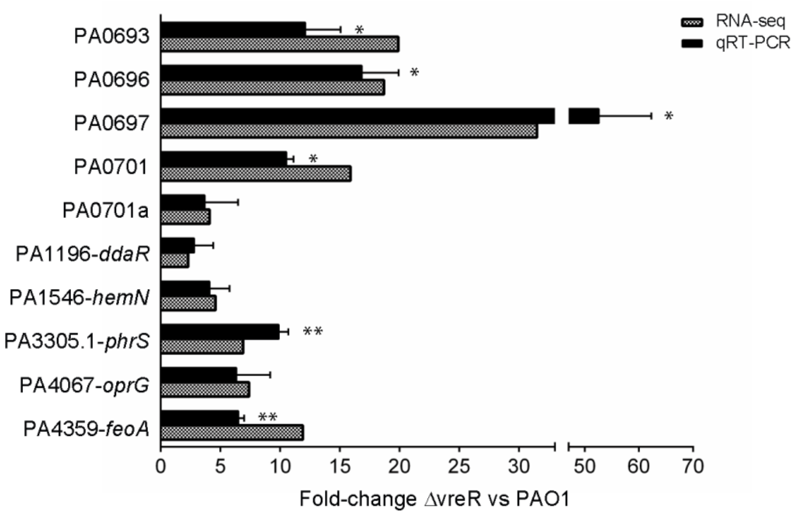

Fig. S1. Comparison between the RNA-seq and qRT-PCR measurements of the differential gene expression in *P. aeruginosa* ΔvreR and PAO1. mRNA levels of the indicated genes were obtained by RNA-seq (Table 1) or qRT-PCR upon growth of the *P. aeruginosa* strains in low Pi medium. The $2^{-\Delta\Delta CT}$ method was used to determine the fold-change range in gene expression in ΔvreR vs PAO1 by qRT-PCR and these data are means ± SD from three biological replicates (N=3) each one including three technical replicates. P-values were calculated by one-sample *t*-test to a hypothetical value of 1 as described in Materials and Methods.

Table S1. Bacterial strains and plasmids used in this study[a]

Strain	Characteristics	Reference
E. coli		
DH5α	*supE44 Δ(lacZYA-argF)U169* ϕ80 *lacZ*ΔM15 *hsdR17* (r_K^- m_K^+) *recA1 endA1 gyrA96 thi1 relA1*; Nal^R	[1]
P. aeruginosa		
PAO1	Wild-type strain	[2]
ΔvreA	Markerless PAO1 null mutant in the *vreA* (PA0674) gene	[3]
ΔvreI	Markerless PAO1 null mutant in the *vreI* (PA0675) gene; the mutation affects the expression of the downstream *vreR* gene	[3,4]
ΔvreR	Markerless PAO1 null mutant in the *vreR* (PA0676) gene	[3]
**Plasmid**		
pMP0690	IncP-based replicon plasmid containing a *P. aeruginosa* P*pdtA::lacZ* transcriptional fusion; Tc^R	[3]
pRS-mCherry	pRSET-B plasmid (Invitrogen) containing a mCherry dsRed with an improved Shine/Dalgarno (SD) sequence to optimize for gene expression in bacteria; Ap^R	[5,6]
pME6031	Broad-host-range plasmid, pVS1 replicon; Tc^R	[7]
pME-mCherry	pME6031 carrying in HindIII a 0.74-Kb from the pRS-mCherry plasmid containing the mCherry dsRed gene; Tc^R	This study
pMP0690mCherry	pMP0690 carrying in XbaI a 0.53-Kb PCR fragment containing the mCherry dsRed gene in between and in the same orientation that the *pdtA* promoter and the *lacZ* gene (*pdtA*::*mCherry* transcriptional fusion); Tc^R	This study
pBBR-PoprF	Plasmid carrying the promoter region of the *P. aeruginosa* PAO1 *oprF* gene; Gm^R	[5]
pSMT3-hsp60-mEos3.1	Source of the mEos3.1green fluorescent protein (*gfp*) gene; Hg^R	[8]
pBBR-mEos3.1	pBBR-PoprF carrying in EcoRI-XbaI a 1.0-Kb PCR fragment containing the mEos3.1 *gfp* gene; Gm^R	This study

[a]Ap^R, Gm^R, Hg^R, Nal^R and Tc^R, resistance to ampicillin, gentamycin, hygromycin, nalidixic acid and tetracycline, respectively.

Table S2. Primers used in the qRT-PCR analyses

***P. aeruginosa* gene**	**Primer name**	**Primer sequence (5'→ 3')**
PA0141	PA0141F	ACTATCCCTATCACACGCGG
	PA0141R	GTGTTCCATGAAGCGCTTGA
PA0200	PA0200F	ATTTCCTCTCCATCCCAGCC
	PA0200R	TCCTCCGTTCCTTCGACTTC
PA0690-*pdtA*	PA0690F2	AGCAGCGGGTCAACCAGCAGTT
	PA0690R	ATCCTGCGGGAGATTCAGCG
PA0691-*pdhA*	PA0691F	GCAGGAACTTGATCAGCGCCTA
	GSP2-0691	CGCCCCCTGCCAATCTTCCTG
PA0692-*tpsB*	PA0692F	CTGGCTGTCGCATTGTCGTG
	PA0692R	CGGTTGTCCAGCACGGTGTT
PA0693-*exbB2*	PA0693F	TGCGGCGATCAACGACAGTC
	GSP2-0693	GGCGGCGACGAAGCGGATGT
PA0696	PA0696F	CTGGGGATGGGCGTCATGCT
	GSP2-0696	CCAGCAGGCGGATCAGGTTG
PA0697	PA0697F1	AGTTGCGCAGCACCACCCAGC
	PA0697R1	CCTGTTGCTGGGCCGAGAG
PA0701	PA0701F	ACGCGACGACCGTGCCTATT
	PA0701R	ATGAAACAGCCGCAACGGG
PA0701a	701-1F	ATTGGCAGGGCGAGGTCT
	701-1R	GTCTCCGCCAGCAGCAACT
PA1196-*ddaR*	PA1196F	GAGGAACCGACGATTTCCAC
	PA1196R	TGTAGTAGACGAAGCGACCG
PA1414	PA1414F	ACGCCGGTCTACTCTTTCCT
	PA1414R	GGTTTTGCAGCCAGAGTTTC
PA1546-*hemN*	PA1546F	GCGTTACACCTCCTATCCGA
	PA1546R	GTAGTAGCAGATGTTGGCGC
PA1556-*ccoO2*	PA1556F	TGTTCTTCCAGGACGTGACC
	PA1556R	AGCCTTCGCGGATATAGATG
PA1673	PA1673F	ATCAACACAAACGCATCGTC
	PA1673R	AGTCCACCAGCTCCTCGAT
PA1746	PA1746F	GTCGATGGTTCGCGACTAC
	PA1746R	GACGATGCACTGGGTCTTG
PA3305.1-*phrS*	PA3305.1F	CTCATGGTCGCTTTCTTCGG
	PA3305.1R	CTTGCGTGCTCTGTGTATCC
PA3337-*rfaD*	PA3337F	GGCGAGACCGACATCATC
	PA3337R	CGTTCTGGTCCAGGTAGTCG
PA3880	PA3880F	GCTGCATTGCGTGAAAGG
	PA3880R	GCCGTAGCGCTTCTTCAG
PA4067-*oprG*	PA4067F	CGCTGCGGATATTCAAGGAC
	PA4067R	TCCAGCTTGATGTCGGAACT
PA4348	PA4348F	CCAACCAGTTCCTGATCGTC
	PA4348R	AAGCGTTTCGACAATTCCAG
PA4359-*feoA*	PA4359F	CATTGCAACCGTCCCGTTC
	PA4359R	AGAAGCCCCATGGAGAACAG
PA5027	PA5027F	ATGATCCGCCACCTCCTG
	PA5027R	TCGTACACGTGCAGCAGACT
PA5304-*dadA*	PA5304F	AGTGCGTATTACCTGGCCC
	PA5304R	TTTCCAGCAGCCACTTCATG
PA5427	PA5427F	CCTGCCACAGACCATGAAAG

	PA5427R	GAGGCTTCGATCTTCACCAG
PA5475	PA5475F	CGACCATTGGGTCGAGTC
	PA5475R	GTTGATGAACTGGCGTTCG
RNAr 16S	13-RNAr 16S	AAAGCCTGATCCAGCCAT
	14-RNAr 16S	GAAATTCCACCACCCTCTACC

Table S3. Other primers used in this study

Plasmid	Primer name	Primer sequence (5’→ 3’)[a]
pMP0690mCherry	mcherryF-X	AAA**TCTAGA**AAGCTTGAGGAGGAGATCCA
	mcherryR-X	AAA**TCTAGA**TTACTTGTACAGCTCGTCC
pBBR-mEos3.1	mEosF-E	GGG**GAATTC**CGACCATTTACGGGTCTTGT
	mEosR-X	CGC**TCTAGA**TATCCATGGATCC

[a]The sequences of the restriction sites are indicated in bold and the annealing region is underlined.

References:

1 Hanahan, D. Studies on transformation of *Escherichia coli* with plasmids. *J Mol Biol* **166**, 557-580 (1983).

2 Jacobs, M. A. *et al.* Comprehensive transposon mutant library of *Pseudomonas aeruginosa*. *Proc Natl Acad Sci U S A* **100**, 14339-14344 (2003).

3 Quesada, J. M., Otero-Asman, J. R., Bastiaansen, K. C., Civantos, C. & Llamas, M. A. The activity of the *Pseudomonas aeruginosa* virulence regulator σ^{VreI} is modulated by the anti-σ factor VreR and the transcription factor PhoB. *Front Microbiol* **7**, 1159 (2016).

4 Faure, L. M., Llamas, M. A., Bastiaansen, K. C., Bentzmann, S. d. & Bigot, S. Phosphate starvation relayed by PhoB activates the expression of the *Pseudomonas aeruginosa* σ^{vreI} ECF factor and its target genes. *Microbiology* **159**, 1315-1327 (2013).

5 Llamas, M. A. *et al.* A novel extracytoplasmic function (ECF) sigma factor regulates virulence in *Pseudomonas aeruginosa*. *PLoS Pathog* **5**, e1000572 (2009).

6 Shaner, N. C. *et al.* Improved monomeric red, orange and yellow fluorescent proteins derived from *Discosoma* sp. red fluorescent protein. *Nat Biotechnol* **22**, 1567-1572 (2004).

7 Heeb, S. *et al.* Small, stable shuttle vectors based on the minimal pVS1 replicon for use in gram-negative, plant-associated bacteria. *Mol Plant Microbe Interact* **13**, 232-237 (2000).

8 Boot, M. *et al.* A fluorescence-based reporter for monitoring expression of mycobacterial cytochrome bd in response to antibacterials and during infection. *Sci Rep* **7**, 10665 (2017).
